# Effects of excimer laser phototherapeutic keratectomy in limbal-conjunctival autograft transplantation for recurrent pterygium: a retrospective case control study

**DOI:** 10.1186/s12886-019-1248-1

**Published:** 2019-11-21

**Authors:** Di Chen, Xiaowei Liu, Qin Long, Zhonghai Wang, Ying Li

**Affiliations:** 1 Department of Ophthalmology, Peking Union Medical College Hospital, Chinese Academy of Medical Sciences & Peking Union Medical College, Beijing, 100005 China; 2 Peking Union Medical College Hospital, Shuaifuyuan 1, Dongcheng District, Beijing, 100730 China

**Keywords:** Recurrent pterygium, Limbal-conjunctival autograft transplantation, Excimer laser phototherapeutic keratectomy, Corneal topography, In vivo confocal microscopy

## Abstract

**Background:**

Repeated surgery excisions could induce obvious irregular astigmatism in patients with recurrent pterygium. Our study is aimed to illustrate the effect of adjunct excimer laser phototherapeutic keratectomy (PTK) in limbal-conjunctival autograft transplantation on visual quality for patients with recurrent pterygium.

**Methods:**

Retrospective case-control study. Eyes that underwent pterygium excision with (PTK group) or without (control group) PTK from 2006 to 2017 were retrospectively included. Recurrence rate, preoperative and postoperative surface regularity index (SRI), surface asymmetry index (SAI), cylinder and LogMAR vision were collected. Postoperative anterior segment optical coherence topography and in vivo confocal microscopy were performed to monitor the cornea epithelium healing and cellular recovery process respectively.

**Results:**

A total of 99 eyes of 99 patients were collected, of which 39 were treated with PTK and 60 without PTK. The mean follow-up time was 50.4 ± 38.1 months. The recurrence rate was 10.3% (4 eyes) in the PTK group and 13.3% (8 eyes) in the control group (*p =* 0.759). The SRI decreased 0.53 (range: − 0.88, 2.81), SAI decreased 0.53 (range: − 0.64, 2.94), and the cylinder decreased 2.08 (range:-0.16, 9.40) D in the PTK group, and the corresponding values were 0.48 (range:-0.45, 2.27), 0.27 (range:-1.06, 2.21) and 0.71 (range:-1.75, 3.55) D in the control group, respectively (*Z* = 1.76, 2.15, and 3.97, *p* = 0.005, 0.016, and 0.000 respectively). LogMAR vision improved in both groups after surgery, with an improvement of 0.18 (range: 0.00, 0.70) in the PTK group and 0.06 (range: − 0.12, 0.50) in the control group (*Z* = 4.08, *p* = 0.000). Besides, the eyes treated with PTK showed faster re-epithelization and better cellular recovery.

**Conclusions:**

For recurrent pterygium, surgical excision with adjunct PTK might be a better option with improved corneal surface and vision outcomes.

## Background

Pterygium is a common chronic ocular surface disease with an overall incidence rate ranging from 1.9 to 9.84% [1, 2]. The only method to eliminate pterygium is surgical removal. However, recurrence is still the most common complication despite a spectrum of adjunct therapies, including conjunctival autograft transplantation with mitomycin C (MMC), cyclosporine, β-irradiation, and hyperbaric oxygen [3–6]. Recurrent pterygium fibrovascular tissue adheres to the anterior corneal stroma firmly. Repeated surgical removal could cause obvious postoperative irregular astigmatism and the recurrence rate could be even higher after secondary operation [7]. Adjuvant in surgery for recurrent pterygium is warranted to achieve a lower recurrence rate, including MMC, 5-fluorouracil (5-FU), amniotic membrane (AM) grafts and conjunctival or limbal autografts [8]. In 1998, Talu H, et al. reported the application of excimer laser phototherapeutic keratectomy (PTK) combined with simple excision to treat recurrent pterygium [6]. Our study compares the clinical outcomes of limbal-conjunctival autograft transplantation (LCAT) with or without adjunct PTK in patients with recurrent pterygium and illustrates the effects of PTK on the corneal surface after repeated pterygium removal.

## Methods

### Subjects

This retrospective case-control study was conducted at the Department of Ophthalmology in the Peking Union Medical College Hospital (PUMCH) and was approved by the Ethic Board of PUMCH (S-K394). Medical records of patients who were diagnosed with recurrent pterygium and received surgical removal and LCAT with or without adjunct PTK from January 2006 to August 2017 were consecutively reviewed. Informed consents were obtained from all subjects.

Inclusion criteria: aged 18 years old or more; recurrent nasal pterygium with corneal invasion of at least 2 mm; patients received surgical removal and LCAT with or without adjunct PTK who had complete medical records and were followed up for more than 1 year.

Exclude criteria: patients with temporal pterygium; patients received amniotic membrane or free conjunctiva flap transplantation without limbus; inadequate follow-up time (less than 12 months).

### Preoperative evaluation

Before surgery, a comprehensive ophthalmic examination was performed, including uncorrected and best corrected visual acuity (BCVA), corneal topography (Tomey TMS-4, Nagoya, Japan), refraction, intraocular pressure, slit-lamp biomicroscopy, and dilated fundus examination. Surface asymmetry index (SAI) and surface regularity index (SRI) were obtained from the topography. Anterior slit-lamp microscopic photography was taken for each patient. Additionally, 0.3% levofloxacin (Santen, Japan) eye drops were prescribed four times daily for 3 days before surgery.

### Surgical procedure

Pterygium excision was performed combined with LCAT under local anesthesia (subconjunctival injection of 2% lidocaine containing 1:100,000 epinephrine). Calipers were used during surgery to measure the corneal invasion length of the recurrent pterygium. The fibrovascular and cicatrix tissues were carefully dissected and removed from the sclera and cornea using a 15 Bard–Parker blade. The corneal surface was polished using the same blade to minimize the residual remnants. A free autologous limbal conjunctival graft containing corneal tissue was measured with calipers and fitted with the conjunctival defect area, then it was harvested from the superior or inferior limbus. While maintaining limbus-to-limbus polarity, the graft was carefully transferred and fixed with interrupted 8–10 stitches. A disposable soft bandage contact lens (Bausch & Lomb) was placed on the cornea at the end of surgery for the patient’s comfort. All the surgeries were done by the same surgeon (Dr. X. Liu) throughout the study.

On the first postoperative day, the patients were evaluated under slit-lamp microscopy and recommended for PTK procedure. Since PTK is not covered by insurance in China, it’s both the doctor’s and patient’s decision to accept the procedure or not. Patients who accepted the PTK procedure were included in the PTK group while those who didn’t accept the procedure were in the control group. For patients in the PTK group, the MEL80 (Carl Zeiss Meditec, Jena, Germany) excimer laser was used to perform corneal ablation with an emission wavelength of 193 nm, an energy fluency of 240 mJ/cm^2^, and a repetition rate of 13 Hz. Initially, all visible residual tissues were ablated in spot mode with a spot of 3 mm. When visible remnants were grossly ablated, a drop of methylcellulose was instilled in the pterygium bed. Slit mode of the excimer laser was used to achieve uniform ablation of the pterygium bed. Generally, a depth of 50 to 100 μm was ablated.

### Postoperative evaluation and data collection

The post-operative regimen included topical tobramycin-dexamethasone solution (Alcon Laboratories, Inc., Texas, USA) 4 times per day for 1 week, and tapered down in 1 month. The patients were assessed at postoperative day (POD)1, 4 and 7; week (POW) 2 and 3; and month (POM) 1,3,6,12 and 18 for BCVA, IOP and slit-lamp microscopy. Anterior segment optical coherence topography (AS-OCT, Visante AS-OCT 1000; Carl Zeiss Meditec, Dublin, CA, USA) was performed in all patients on POD4 to assess the healing of corneal epithelium and repeated daily until the whole corneal was re-epithelized. Recurrence, in vivo confocal microscopy (IVCM, Heidelberg Retinal Tomograph with Rostock Corneal Module, Heidelberg Engineering, GmBH, Dossenheim, Germany) and corneal topography were evaluated every 3 months during the follow-up. LogMAR improvement (ΔLogMAR) was defined as the difference between preoperative and postoperative (6 months) LogMAR visual acuity values. SAI/SRI decrement (ΔSAI/ΔSRI) was defined as the difference between preoperative and postoperative (6 months) SAI/SRI values, and cylinder decrement (Δcylinder) was defined as the difference between preoperative and postoperative (6 months) cylinder values. Recurrence was defined as fibrovascular proliferative tissue crossing the limbus in this study [9].

### Statistical analysis

SPSS version 16.0 (SPSS Inc., Chicago, Illinois, USA) was applied for the statistical analysis. The data were given as mean ± standard deviation (SD) and compared with Student’s t-test for normally distributed parameters (age, corneal invasion length and recurrence interval). For the parameters that were not normally distributed (ΔLogMAR, ΔSAI, ΔSRI and Δcylinder), the data were displayed as median (range) and compared with Mann-Whitney U test. Chi-square test or fisher’s exact test was used for the analysis of categorical data. *P* < 0.05 was considered statistically significant.

## Results

A total of 99 eyes of 99 patients were eligibly accessed in this study (Table 1). The right eye was included if a patient had recurrent pterygium in both eyes (11patients). There was no difference in age (*t* = 1.17, *p* = 0.24) or sex ratio (χ^2^ = 1.044, *p* = 0.307) between two groups. The mean follow-up time was 50.4 ± 38.1 months. No differences were found between two groups regarding the number of previous pterygium excision surgeries before this study (Table 1). The last pterygium surgery occurred at least 6 months before study.

**Table 1:** Demographics of Subjects

	PTK (*N* = 39)	Control (*N* = 60)	*p* value
Age (mean ± SD)	55.1 ± 10.1	57.5 ± 9.9	0.240*
Sex (male/female)	15/24	23/37	0.307^#^
Number of previous pterygium surgeries			
1	35.9% (14/39)	45.0% (27/60)	0.359^†^
2	38.5% (15/39)	41.7% (25/60)	
3	17.9% (7/39)	11.7% (7/60)	
4	7.7% (3/39)	1.7% (1/60)	

* two-tailed t test; ^#^Chi-square test; ^†^ Fisher’s exact test

*p*-values less than 0.05 are considered significant

*PTK* Excimer laser phototherapeutic keratectomy

**Fig. 1:**
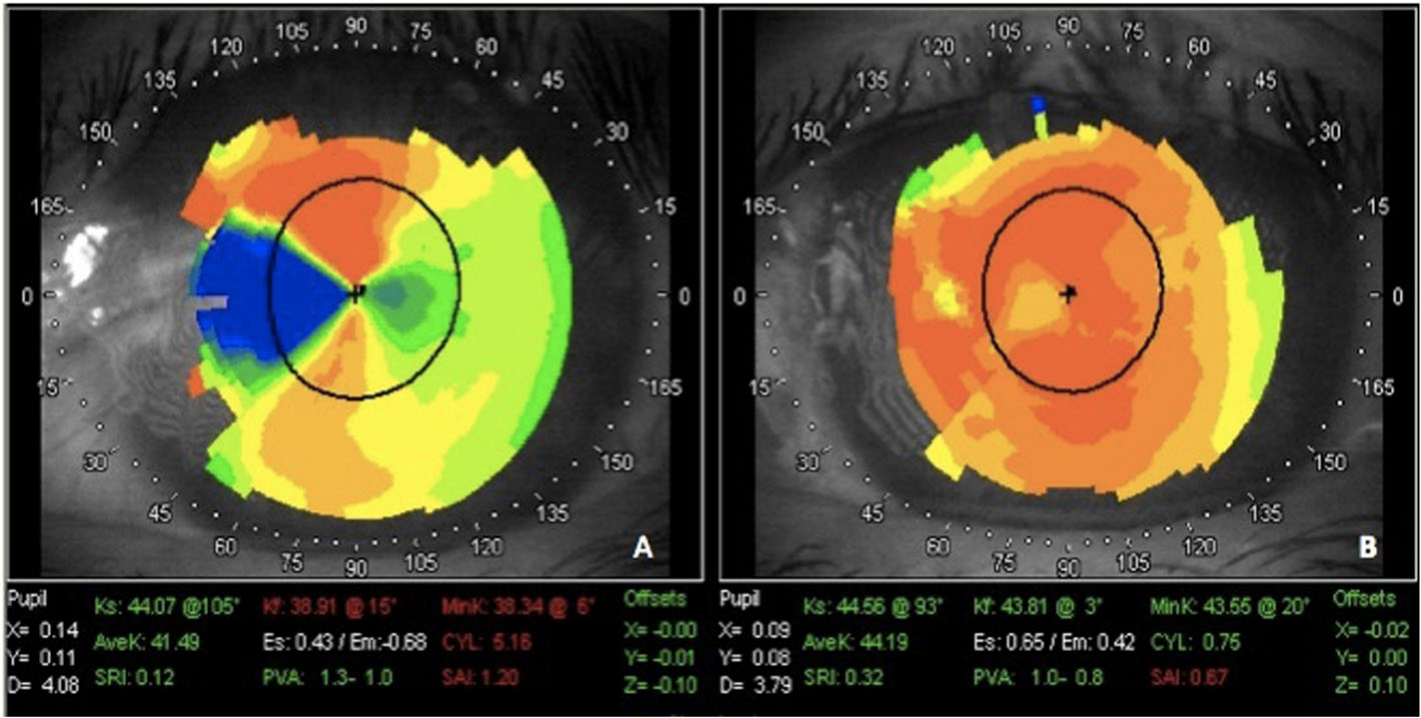
Corneal topography changes before and after recurrent pterygium excision and PTK treatment. A50-year-old female suffering from recurrent pterygium in her left eye with prominent irregular astigmatism (**a**). Two weeks after surgical excision and PTK treatment, a relatively smooth corneal surface with significant reduction in cylinder was found on corneal topography (**b**). PTK: excimer laser phototherapeutic keratectomy

**Fig. 2:**
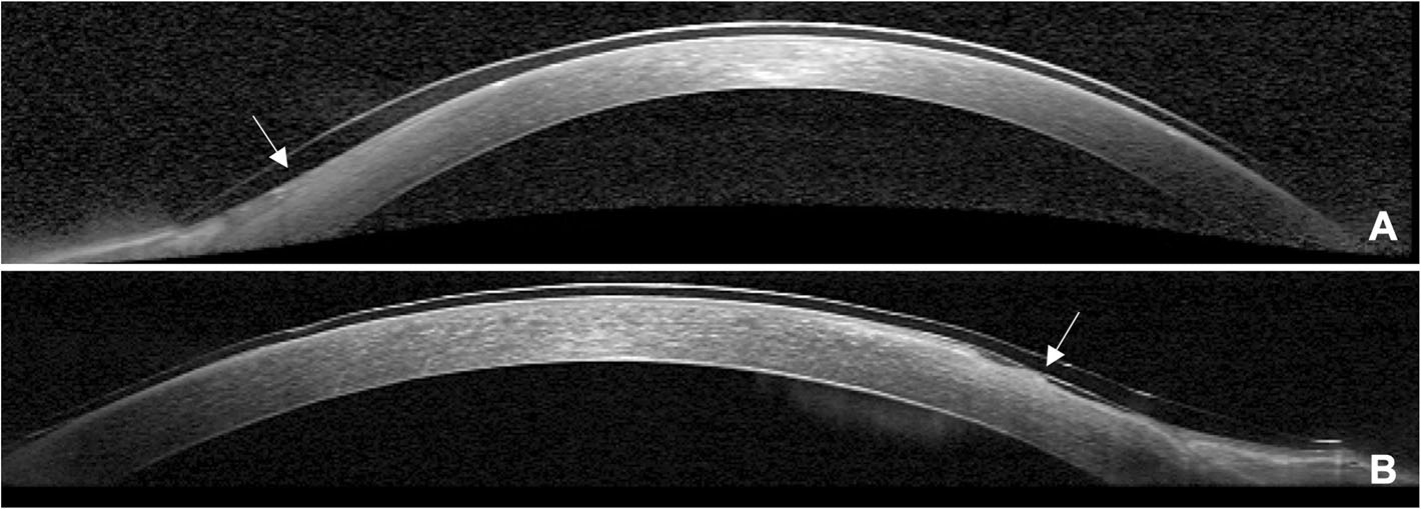
Anterior segment optical coherence topography images of patients. **a** A smooth corneal surface and intact epithelium (white arrow) were observed beneath the contact lens in a 56-year-old female received PTK on postoperative day 4; **b** A rough surface with stromal pits and a defect in the epithelial (white arrow) layer under the contact lens in a 58-year-old female who didn’t receive PTK treatment. PTK: excimer laser phototherapeutic keratectomy

Table 2 summarized the clinical outcomes of two groups. The corneal invasion length in PTK group was even longer than the control before surgery (*p* = 0.006). Both treatments showed an improvement in visual acuity and corneal topography, and the PTK group displayed significantly more improvement compared with the control group (Table 2, Fig. 1). Furthermore, smoother corneal surfaces and more intact epithelium were observed in the PTK group. Local abruption of the epithelium layer and rough ocular surfaces were observed in some cases in control group. A stromal pit was formed and remnants of the fibrous tissue over the corneal stroma were revealed by AS-OCT (Fig. 2).

**Table 2:** Comparison of clinical outcomes between PTK and control group

	PTK (*N* = 39)	Control (*N* = 60)	*p* value
Corneal invasion length	4.2 ± 1.12	3.49 ± 1.26	0.006*
ΔLogMAR	0.18 (0.12,0.38)	0.06 (0.00,0.18)	**0.000**^**§**^
ΔSAI	0.53 (0.22,2.31)	0.48 (0.22,0.60)	**0.005**^**§**^
ΔSRI	0.53 (0.07,1.61)	0.27 (0.08,0.59)	**0.016**^**§**^
ΔCylinder	2.08 (0.76,3.09)	0.71 (0.22,1.56)	**0.000**^**§**^
Unhealed epithelium on POD4	2.56% (1/39)	13.3% (8/60)	0.084^†^
Under-graft bleeding	7.69% (3/39)	3.33% (2/60)	0.380^†^
Graft edema	2.56% (1/39)	1.67% (1/60)	0.628^†^
Recurrence rate	10.3% (4/39)	13.3% (8/60)	0.759^†^
Recurrence interval (month)	3.13 ± 2.68	2.88 ± 1.86	0.590*

* two-tailed t test; ^**§**^ Mann-Whitney U test; ^†^ Fisher’s exact test

For corneal invasion length and recurrence interval, data were displayed as mean ± SD; for ΔLogMAR, ΔSAI, ΔSRI, ΔCylinder, data were displayed as median (range); for unhealed epithelium on POD4, under-graft bleeding, graft edema and recurrence rate, data were displayed as percentage (number). *p*-values less than 0.05 are considered significant and highlighted in bold

*PTK* Excimer laser phototherapeutic keratectomy, *SAI* Surface asymmetry index, *SRI* Surface regularity index, *POD* Postoperative day, ΔLogMAR was defined as the difference between preoperative and postoperative (6 months) LogMAR visual acuity; ΔSAI/ΔSRI was defined as the difference between preoperative and postoperative (6 months) SAI/SRI; Δcylinder was defined as the difference between preoperative and postoperative (6 months) cylinder

**Fig. 3:**
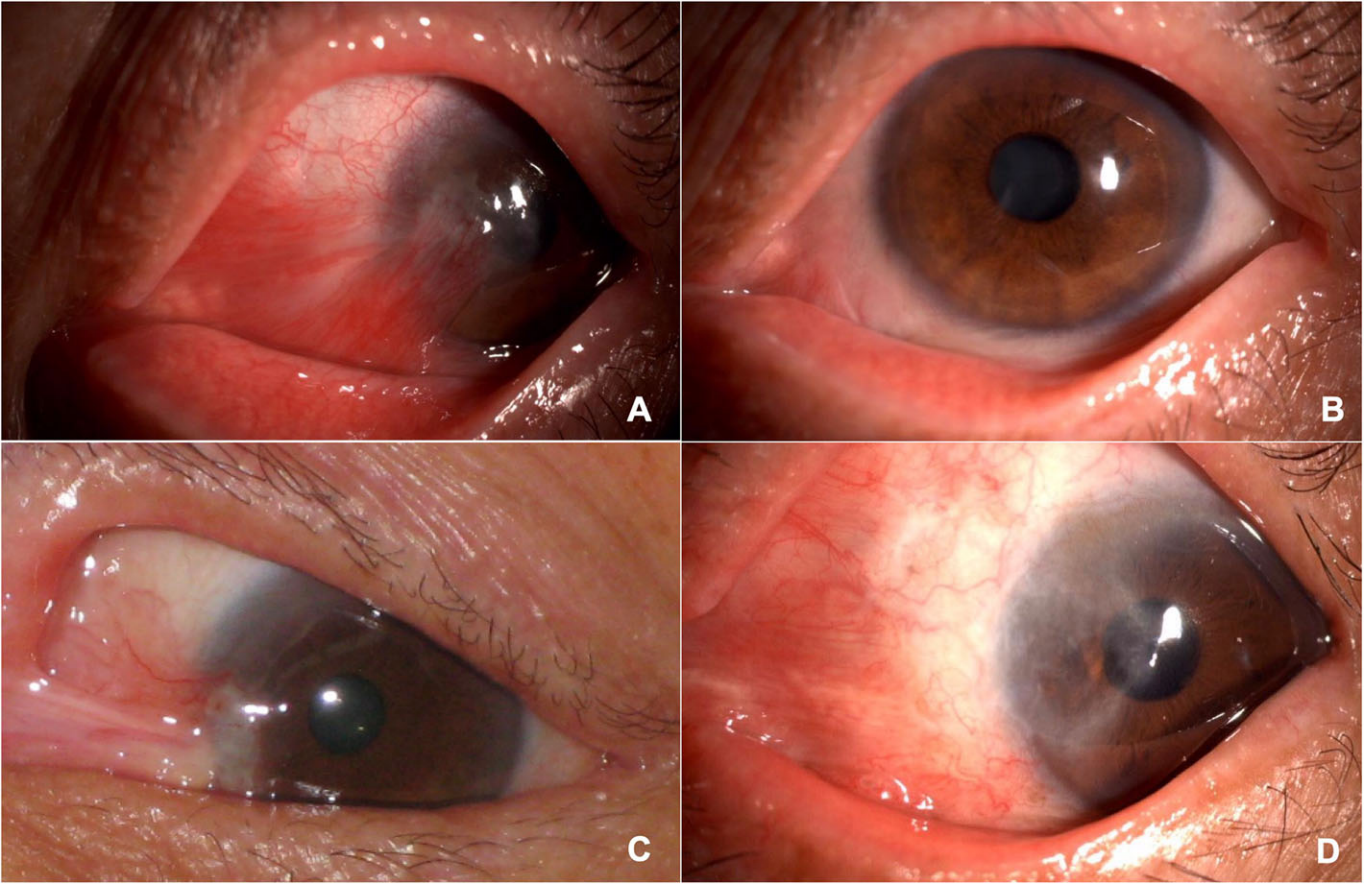
Slit-lamp photos of the patients. **a** Preoperative recurrent pterygium affecting the pupil area in a 63-year-old male before surgery. **b** Six months after surgical removal and PTK treatment, the cornea was clear and smooth. **c** Recurrent pterygium in a 67-year-old male. **d** Six months after surgery without PTK treatment, the nasal cornea was rough and opaque. PTK: excimer laser phototherapeutic keratectomy

**Fig. 4:**
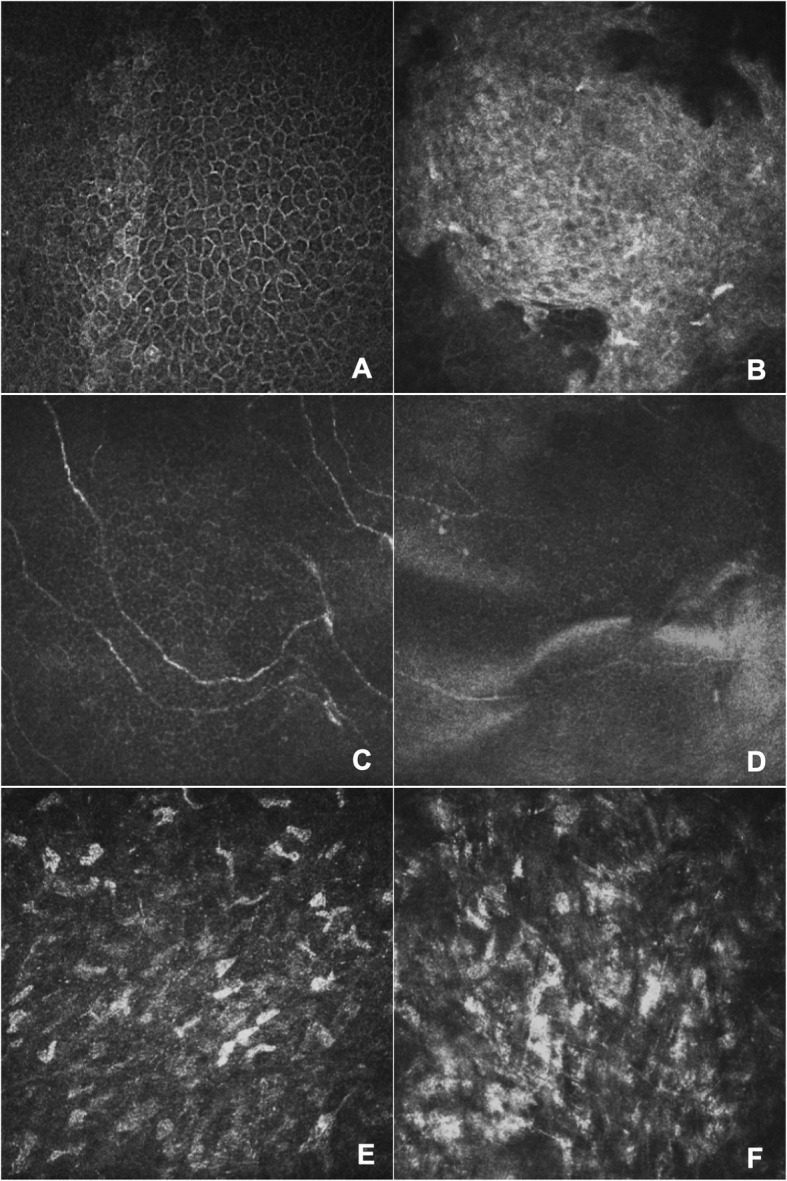
In Vivo Confocal Microscopy examination of the patients 6 months after surgery with (**a**, **c**, **e**) or without (**b**, **d**, **f**) PTK treatment. **a** The epithelium exhibited slightly irregular polygonal basal cells in the surgical area with some brightly reflective cell structures. **b** Highly reflective masses with irregular cell shapes were observed. **c** Normal (in length and density) subepithelial nerve plexus. **d** Decreased nerve density and short nerve fibers. **e** Normal stromal cells with mild bright scar tissue; **f** Highly reflective scar tissue in the stroma

The patients treated with PTK exhibited clearer and smoother cornea in appearance (Fig. 3), which was also confirmed by IVCM (Fig. 4). IVCM showed slightly irregular polygonal basal cells in the surgical area with limited brightness reflecting cell structures (Fig. 4a) and a relatively smooth cell layer in PTK group. In the control group, the epithelial cells were more irregular, and the cell structure could not be visualized, possibly due to the rough and uneven surface and wing cells, basal cells and scar fibers in the same layer (Fig. 4b). The sub-epithelial nerve plexus was almost normal in length and density in the PTK patients under IVCM, except for a mildly distorted border between the normal and the surgical area (Fig. 4c). In the control group, nerve fiber density decreased, and full-length fibers were not easily visualized, indicating a rough surface layer (Fig. 4d). The anterior stroma appeared normal with mild, bright scar tissue (Fig. 4e) in PTK group and highly reflective scar tissue in the controls (Fig. 4f).

Recurrence was identified in 4 eyes (10.3%) in the PTK group and in 8 (13.3%) eyes in the control group, which were treated with weekly 5-FU intralesional injections. All eyes became quiescent and stable. No significant differences in the recurrence rate (*p =* 0.759) or the recurrence time (*p* = 0.59) were found between the two groups. PTK treatment didn’t induce extra graft bleeding or edema compared with controls (Table 2). No infections, corneal ulcers or corneal melting were observed during the follow-up period.

## Discussion

Pterygium excision and the bare sclera technique for recurrent pterygium have been abandoned in recent years because of the high recurrence rate [10]. Pterygium excision with LCAT is considered effective for the treatment of primary and recurrent pterygium to reduce the recurrence rate [10, 11], and it also appears to be a better option compared with conjunctival autograft without limbus and amniotic membrane transplantation [11–13]. MMC, beta irradiation [8, 14], hyperbaric oxygen [3] and anti-VEGF [15] may also further decrease the recurrence rate, but the associated vision-threatening complications are concerning [6, 16]; these adjunct therapies are controversial and are not available in most hospitals in China.

In recurrent pterygium, fibrovascular and scar tissues adhere to the stroma and sclera firmly. After initial excision, a rough surface with a stromal pit and remnants of the fibrous tissue are generally inevitable and can cause severe irregular astigmatism [17]. Lamellar keratoplasty was typically required to improve vision [18]. Excimer laser PTK can smooth the corneal surface and ablate residual tissues and stromal scars better than the diamond fraise technique without thermal damage to the nearby corneal tissue, preventing scar formation and neovascularization [19]. Excimer laser PTK appears to be a helpful adjunct procedure in recurrent pterygium surgery to achieve better visual outcomes [6]. In this study, smoother corneal surfaces were confirmed by slit-lamp examination, corneal topography, AS-OCT and IVCM. Furthermore, PTK could facilitate cornea re-epithelization after pterygium excision [20]. A smooth corneal surface provides a better basement membrane for the corneal epithelium to attach and spread, which was confirmed by AS-OCT examination in this study. PTK combined with refractive correction could also be performed several weeks or months following pterygium removal. However, POD1 was set as the time of PTK in this study to avoid repeated cornea epithelium removal.

Corneal topography is a useful and powerful tool for evaluating the refractive changes caused by pterygium [21]. SAI, SRI and cylinder are the most important values in pterygium patients. Pterygium could cause the flattening of the central cornea, and recurrent pterygium may induce significant corneal distortion, causing the SRI, SAI and cylinder to increase dramatically [17]. After pterygium excision, these parameters improve gradually and may become stable over a period of approximately 3 months. The preoperative parameters were not comparable between the two groups, and the patients in the PTK group had higher SRI, SAI and cylinder values than the controls. Therefore, we compared the changes of SRI, SAI and cylinder before and after surgery between the two groups and found that the patients in the PTK group showed more obvious SRI, SAI and cylinder improvements after surgery than the controls. The smooth corneal surface achieved with PTK can help patients obtain more vision improvements. A smooth corneal surface and less scar tissue on the cornea after PTK treatment showed that this approach might be a better and safer choice than lamellar keratoplasty [22]. However, the corneal topographic data were collected from the center or para-center of the cornea, a small and thin pterygium invading into the cornea within 2–3 mm could cause tremendous limbal changes but only mild central corneal parameter changes. To avoid these biases, better tools to evaluate pterygium corneal surface changes should be investigated.

The speed of corneal re-epithelization depends on the basement membrane. A smooth corneal surface achieved by PTK provides a better basement membrane for new epithelium to attach and spread than that observed in the untreated controls. Usually, fluorescein staining has been used to test corneal epithelium healing, but in this study, to relieve postoperative pain, foreign body sensation, tearing and photophobia, we covered the corneal epithelial defect with a bandage contact lens. Therefore, AS-OCT was used to monitor epithelial healing in this study to avoid disturbing re-epithelization or peeling of the unattached new epithelium or potential contamination by fluorescein [23]. A smoother ocular surface and a shorter healing time were observed via OCT in PTK-treated eyes.

IVCM can reveal the microscopic cell structures of different layers of the cornea. Recurrent pterygium removal inevitably damages the epithelium, Bowman’s layer, subepithelial nerve plexus and anterior stroma [24]. In this study, IVCM provided more details regarding cell structure related to the efficacy of PTK treatment in reducing corneal scar formation, decreasing remnants, and smoothing the corneal surface. To our best knowledge, this is the first report on IVCM following pterygium removal for PTK-treated corneas. In this study, the epithelium, nerve and stroma showed greater improvements in the PTK group than in the controls.

PTK could help patients obtain a satisfactory refractive corneal surface and reduce the recurrence rate [6, 25]. A 12.5% recurrent rate was reported for recurrent pterygium excision, and a rate of 0.02% was reported for MMC use in surgery [26]. In this study, we found a similar recurrence rate of 10.3% in the PTK-treated patients and 13.3% in the controls. However, the preoperative values of LogMAR BCVA, SRI, SAI, cylinder and corneal invasion length were significantly higher in the PTK group than the controls. Unbalanced baseline values may explain the insignificant recurrence rates between the two groups in this study. Talu H et al. reported a group of 22 recurrent pterygium patients treated with excision, PTK and the bare sclera technique and found a recurrence rate of 4.5% [6]. However, in their study, no controls were included, and the preoperative clinical data may be different from our patients.

In most hospitals and clinics in China, excimer lasers are mounted in specialized operation rooms (ORs), while the pterygium removal surgery is performed in a regular OR. Patients may have to be referred to another doctor for PTK treatment on a different day, which may impede the implementation of PTK after pterygium removal in China.

This study has some limitations. This was not a prospective, randomized study. Selection bias cannot be excluded. Patients with a good economic status are likely to accept PTK which is expensive and is not covered by insurance in China. In this study, less than half of the patients accepted PTK surgery following our recommendation. Therefore, a randomized study should be considered in the future to confirm our findings.

## Conclusions

In this study, we compared recurrent pterygium patients who underwent pterygium removal and LCAT with or without PTK treatment. Although the recurrence rates were not significantly different, the patients treated with PTK after recurrent pterygium removal showed greater BCVA, SRI, SAI and cylinder improvements, a smoother corneal surface, faster re-epithelization, and better cellular recovery based on IVCM. PTK could be another choice of adjunctive therapy for recurrent pterygium surgery with an acceptable recurrence rate.

## Data Availability

Data and materials related to this work are available from the corresponding author upon reasonable request.

## Abbreviations

AS-OCT: Anterior segment optical coherence topography
BCVA: Best corrected visual acuity
IVCM: In vivo confocal microscopy
LCAT: Limbal-conjunctival autograft transplantation
OR: Operating room
POD: Postoperative day
PTK: Limbal-conjunctival autograft transplantation
SAI: Surface asymmetry index
SRI: Surface regularity index
